# Supplementary material for: Establishment of dry-chemistry-based reference intervals of routine liver function tests for the adult population of Gandaki Province, Nepal
Source: PLOS Glob Public Health. 2024 May 9;4(5):e0001865. doi: 10.1371/journal.pgph.0001865 (PMC11081272; doi:10.1371/journal.pgph.0001865)
Supplement: Attachment — Submitted filename: Response to Reviewer.pdf [file pgph.0001865.s005.pdf]

Section	Comments	Respond
Journal requirement	We ask that a manuscript source file is provided at Revision. Please upload your manuscript file as a .doc, .docx, .rtf or .tex.	The revised manuscript file has been uploaded in the editorial manager's webpage.
	We do not publish any copyright or trademark symbols that usually accompany proprietary names, eg ©, ®, TM (e.g. next to drug or reagent names). Please remove all instances of trademark/copyright symbols throughout the text, including ® on pages 7 & 8.	The copyrights or trademark symbols mentioned in the manuscript have been removed.
	We have noticed that you have uploaded Supporting Information files, but you have not included a list of legends. Please add a full list of legends for your Supporting Information files after the references list.	The full list of legends has been added to the supporting information file.
	We notice that your supplementary files are included in the manuscript file. Please remove them and upload them with the file type 'Supporting Information'. Please ensure that each Supporting Information file has a legend listed in the manuscript after the references list.	The supplementary file has been removed from the main body of the manuscript and uploaded separately as Supporting Information.

	In the online submission form, you indicated that "The datasets generated and analyzed during the current study would be available from the corresponding author on personal request". All PLOS journals now require all data underlying the findings described in their manuscript to be freely available to other researchers, either 1. In a public repository, 2. Within the manuscript itself, or 3. Uploaded as supplementary information. This policy applies to all data except where public deposition would breach compliance with the protocol approved by your research ethics board. If your data cannot be made publicly available for ethical or legal reasons (e.g., public availability would compromise patient privacy), please explain your reasons by return email and your exemption request will be escalated to the editor for approval. Your exemption request will be handled independently and will not hold up the peer review process, but will need to be resolved should your manuscript be accepted for publication. One of the Editorial team will then be in touch if there are any issues.	We have presented all processed data in the manuscript and supplementary information file, except for the data that reveals the identity of the study participants. We are unable to provide complete raw data with the study participants' identities as it would violate the protocol approved by our institutional research and ethics committee and the commitment expressed in the written informed consent. Therefore, We would like to request the editor to waive this requirement for us. If required, we would be writing a separate request mail to the journal editor.

**Reviewer 1**	The authors need to justify Why population-specific references are needed especially for Nepalese population. If everyone establishes their population specific intervals, the entire medical research must be repeated in every population which is not feasible. Where is the end to this? The authors need to comment on this and discuss.	• Reference intervals are important for interpreting laboratory test results and clinical decision-making.They represent our understanding of physiology and the way it normally affects laboratory tests.While devising ranges for every group might appear impractical, it's essential for precision in diagnoses and treatments. Yet, it's unnecessary to conduct full-scale medical studies each time. Instead, current data can be leveraged to set suitable reference intervals. Organizations like the Clinical and Laboratory Standards Institute (CLSI) offer guidance on this front.<br>• In our case, although the method of dry chemistry-based analysis was there in the global diagnostic market for more than 30 years, the OCD Vitros based dry chemistry-based platforms were introduced in Nepal only in 2018. The reference range for biochemical parameters including liver function tests has not been determined for the Nepalese population. Moreover, RIs for this platform is not widely available in scientific literatures and clinical chemistry textbooks.<br>• Next, all RI-related studies are basically meant for a specific lab or population rather than for global application. This is the reason why international laboratory guidelines such as CLSI and IFCC recommend that every laboratory should establish its own RIs. This is because measured biochemical values are not only influenced by age, sex, and lifestyle, but also by the method of estimation, race, dietary habits, and ecological factors like climate, and altitude. Hence, all RI-related studies have local rather than global importance. However, this does not mean that they have no global importance. This is because laboratory of other regions can compare their RI values with our values and also may

		adapt in case, they have not determined them yet and have similar nature of serving the population.<br>• Additional details are added in introduction paragraph.
	The participants are considered as healthy based on the questionnaire and self-declaration which was also mentioned in the limitations. This was acceptable if the derived reference ranges were similar to the global ranges. Since the proposed ranges for this population are different, the participants should have been clinically tested and determined healthy. Attest those participants who are determined as "out of range" must be clinically examined and declared as healthy if these values have to be acceptable.	In our limitation portion we have attested about the population selection.<br><br>We acknowledge and appreciate the reviewer's concern regarding the health status assessment of the participants in our study. We agree that clinical testing would have been an ideal way to ensure the health status of the participants, but we would like to provide further context to explain our approach and constraints.<br><br>The individuals included in our study consisted of visitors and volunteers, and they were participating voluntarily in our research. Due to time limitations and logistical constraints, it was challenging to conduct comprehensive clinical tests on each participant. Moreover, financial limitations posed a significant constraint on the scope of our study. It's important to note that our research project was conducted as a partial fulfillment of a graduate degree, and while we strived for rigor and accuracy within our means, our resources were limited.<br><br>To mitigate these limitations, we designed our study carefully, incorporating a rigorous questionnaire and self-declaration process. We recognize that this self-assessment approach has its limitations, as outlined in our manuscript's limitations section. However, we believe that by filtering out outliers and individuals who did not meet our selection criteria, we were able to minimize the inclusion of individuals with underlying health issues or those who were not truly representative of the healthy Nepalese population.

		While clinical testing for each participant would have been ideal, the constraints we faced made it unfeasible. Nonetheless, we ensured that our data collection and analysis processes were as thorough as possible given the circumstances. We hope that the outcomes of our study, including the derived reference ranges, provide valuable insights despite these limitations. We are open to further discussion and collaboration to address any remaining concerns and improve the robustness of our research in future studies.<br><br>We sincerely appreciate the reviewer's feedback and the opportunity to address these important coniderations.
	Since the authors are proposing the derived values in this study as references for the entire Nepalese population, they should have participants from more than one center across the country.	We agree with the argument of the reviewer in general. However, conducting a study across multiple centers can be resource-intensive in terms of funding, time, personnel, and logistics regarding our context. This study is the pioneer study to represent the reference interval for the Nepalese Population as population from our center were included from 11 districts in Gandaki Province which has a similar inclusion criterion as that of the whole nation. A single-center study can serve as an initial step in deriving reference ranges. Once these preliminary values are established, they can then be validated or refined through larger, multicenter studies if resources and circumstances allow.
	At several places in the manuscript, the references are giving an error "Error! Reference source not found" This has to be rectified.	The references giving such errors have been rectified.

Some changes have been made to the manuscript. The information that hasn't been changed is marked with black colour, with subtle changes made in languages (paraphrased) highlighted in orange and newly added details marked as red.
