# Supplementary material for: Establishment of dry-chemistry-based reference intervals of routine liver function tests for the adult population of Gandaki Province, Nepal
Source: PLOS Glob Public Health. 2024 May 9;4(5):e0001865. doi: 10.1371/journal.pgph.0001865 (PMC11081272; doi:10.1371/journal.pgph.0001865)
Supplement: Attachment — Submitted filename: Comments to second reviewer.pdf [file pgph.0001865.s006.pdf]

Section	Comments	Respond
Journal requirement	Please review your reference list to ensure that it is complete and correct. If you have cited papers that have been retracted, please include the rationale for doing so in the manuscript text, or remove these references and replace them with relevant current references. Any changes to the reference list should be mentioned in the rebuttal letter that accompanies your revised manuscript. If you need to cite a retracted article, indicate the article's retracted status in the References list and also include a citation and full reference for the retraction notice.	Our reference list is complete and we have not cited any papers that have been retracted.
	Some material included in your submission may be copyrighted. According to PLOS's copyright policy, authors who use figures or other material (e.g., graphics, clipart, maps) from another author or copyright holder must demonstrate or obtain permission to publish this material under the Creative Commons Attribution 4.0 International (CC BY 4.0) License used by PLOS journals. Please closely review the details of PLOS's copyright requirements here: PLOS Licenses and Copyright. If you need to request permission from a copyright holder, you may use PLOS's Copyright Content Permission form.	Figure 1 used in our manuscript was created by merging two separate maps (a map of Nepal and a map of Gandaki Province).<br><br>The map of Nepal was adapted from Goodhand J et al 2021 https://www.researchgate.net/publication/352155539_Marginal_gains_borderland_dynamics_political_settlements_and_shifting_centre-periphery_relations_in_post-war_Nepal/figures?lo=1<br><br>and is available freely to copy, adapt and share as per the Creative Commons Attribution 4.0 International (CC BY 4.0) License (https://creativecommons.org/licenses/by/4.0/).<br><br>The map of Gandaki Province was adapted with permission from the news article published in the Kathmandu Post on July 6, 2018 (Link: https://kathmandupost.com/national/2018/07/06/province-4-named-gandaki-pokhara-capital)

	Fig 1: please (a) provide a direct link to the base layer of the map (i.e., the country or region border shape) and ensure this is also included in the figure legend; and (b) provide a link to the terms of use / license information for the base layer image or shapefile. We cannot publish proprietary or copyrighted maps (e.g. Google Maps, Mapquest) and the terms of use for your map base layer must be compatible with our CC-BY 4.0 license. Note: if you created the map in a software program like R or ArcGIS, please locate and indicate the source of thebasemap shapefile onto which data has been plotted.	A webpage link to both sources has been included in the figure legend. Moreover, the link to the terms of use/license information for the main map image has been provided.
**Reviewer 2**	The research consists of 11 parameters commonly used in LFTs. Why selecting only these 11 factors?	We selected these 11 LFT parameters based on their comprehensive coverage of diverse liver functions and hepatocellular injury, and well-established clinical significance in the diagnosis and monitoring of liver diseases. We also selected them because they are the commonly ordered tests in clinical practice to ensure the practicality and applicability of our reference intervals. This approach also ensures that our findings are directly relevant to the tests most commonly used by clinicians.
	The normal weight for BMI is about 18.5 to 24.9 while in this study it is between18.5-27.5. Please reintroduce this selected range.	We used the WHO BMI classification recommended for the Asian population because the participants in our study were South Asians. With a BMI (kg/m^2) of >18.5 is underweight, 18-22 is normal, 23-24 is overweight, 25-29 is obese I, and ≥30 is obese II. We followed the list of selection criteria mentioned in the CSLI/IFCC Document C28-A3, which does not require a normal BMI (18.5-22.9) as a strict inclusion criterion. Furthermore, limiting our study to adult participants with only normal BMI will exclude many qualified participants from participating in our study and will not accurately reflect the general health service-seeking population. Therefore, we raised the

		upper limit of BMI to 27.5 to broaden the scope of our study.
	The range of age for participants is 18-70 years, what is the main reason for that?	This study aimed to establish clinically relevant reference intervals for a diverse adult population aged 18-70, representing those likely to undergo regular liver function testing in clinical practice. This age range aligns with the distribution of individuals seeking medical care and undergoing such testing, enhancing the practical utility of the reference intervals for healthcare providers. The inclusivity of participants across different stages of adulthood allows for the consideration of potential variations in liver function parameters across the adult lifespan.
	What about the association of BMI and age with this result?	Age and BMI are important confounders that might alter the reference intervals of some LFT parameters. Establishing age-group and BMI-specific reference intervals for LFT parameters may be necessary to ensure an accurate interpretation of results. However, this requires a large sample size to adequately represent different age and BMI groups. While several studies have compared LFT parameters across different age and BMI categories, the sample size of our study was not sufficient to establish the BMI and age-specific RIs.

	Participants were checked for a set of medical history-centered questionnaires, general physical examination, and<br>certain screening blood tests which made general screening, while there is some question in following lines.<br>• The fatty liver is a main factor in this screening or not?<br>• Which method/methods was/were used for diagnosing of fatty liver?<br>• Has the lifestyle of participants any association with results?<br><br>• What about who used any kinds of alcohol? Were they checked by questionnaire? And did they leave the study or not?	Our study participants were screened for the possible presence of serious diseases that could potentially confound the results of our study. Their medical history and general health status were screened initially using a set of structured questionnaires followed by a general physical examination by the physician. Any participants clinically diagnosed with chronic liver diseases or fatty liver were excluded from the study. Hence, there was hardly any need for deep screening of participants for fatty liver using techniques like USG. We also collected lifestyle-related data such as dietary, smoking, and drinking habits but could not establish their significant association with the newly established RIs. Participants who used to drink alcohol (locally brewed alcohol, wine, beer, rum, or whisky) regular basis were excluded from the study and only occasional drinkers at long gaps or teetotalers were included in the study.<br><br>Since our study was cross-sectional in nature, we did not encounter the issue of dropping out of the participants in the midway of our study.
	It seems to be suitable adding an inclusion and exclusion chart to manuscript for this study.	The inclusion and exclusion criteria for our study participants have now been presented in the tabulated chart in the manuscript.

	Moreover, The Following reference can be included in the introduction part for more readability:<br>• Age-specific reference intervals for liver function tests in healthy neonates, infants, and young children in Iran. https://doi.org/10.1002/jcla.24995<br>• Age-specific reference intervals for routine biochemical parameters in healthy neonates, infants, and young children in Iran. https://doi.org/10.1111/jcmm.17646<br>• Reference intervals for routine biochemical markers and body mass index: A study based on healthcare center database in northeastern Iran. https://doi.org/10.1002/iub.2437<br>• Pediatric reference intervals for hematology parameters in healthy infants and young children in Iran. https://doi.org/10.1111/ijlh.14132<br>• Interaction between the genetic variant of rs696217-ghrelin and food intake and obesity and dyslipidemia.<br><br>https://doi.org/10.1111/ahg.12443	We carefully considered all of the references suggested to be included in our manuscript and reached the conclusion that only one reference could best fit to be cited in our manuscript as all other references are related to pediatric age groups. Our research focused only on the adult age group participants. Hence, we selected only one reference (https://doi.org/10.1002/iub.2437) and cited in the introduction part of our manuscript.
